# Ensembles of human myosin-19 bound to calmodulin and regulatory light chain RLC12B drive multimicron transport

**DOI:** 10.1016/j.jbc.2023.102906

**Published:** 2023-01-13

**Authors:** Luther W. Pollard, Stephen M. Coscia, Grzegorz Rebowski, Nicholas J. Palmer, Erika L.F. Holzbaur, Roberto Dominguez, E. Michael Ostap

**Affiliations:** 1 Department of Physiology, University of Pennsylvania Perelman School of Medicine, Philadelphia, Pennsylvania, USA; 2 Pennsylvania Muscle Institute, University of Pennsylvania Perelman School of Medicine, Philadelphia, Pennsylvania, USA; 3 Cell and Molecular Biology Graduate Group, University of Pennsylvania Perelman School of Medicine, Philadelphia, Pennsylvania, USA; 4 Biochemistry and Molecular Biophysics Graduate Group, University of Pennsylvania Perelman School of Medicine, Philadelphia, Pennsylvania, USA

**Keywords:** myosin-19, Myo19, myosin-XIX, calmodulin, regulatory light chain, calcium, mitochondrial dynamics, actin gliding, mass spectrometry–based proteomics, Qdot, processivity, CaM, calmodulin, CV, column volume, GraFix, fixation during gradient centrifugation, HC, heavy chain, LC, light chain, MALS, multiangle light scattering, MBP, maltose-binding protein, MD, motor domain, MS, mass spectrometry, Myo19, Myosin-19, Qdot, quantum dot, RLC, regulatory light chain, TEV, tobacco etch virus

## Abstract

Myosin-19 (Myo19) controls the size, morphology, and distribution of mitochondria, but the underlying role of Myo19 motor activity is unknown. Complicating mechanistic *in vitro* studies, the identity of the light chains (LCs) of Myo19 remains unsettled. Here, we show by coimmunoprecipitation, reconstitution, and proteomics that the three IQ motifs of human Myo19 expressed in Expi293 human cells bind regulatory light chain (RLC12B) and calmodulin (CaM). We demonstrate that overexpression of Myo19 in HeLa cells enhances the recruitment of both Myo19 and RLC12B to mitochondria, suggesting cellular association of RLC12B with the motor. Further experiments revealed that RLC12B binds IQ2 and is flanked by two CaM molecules. *In vitro*, we observed that the maximal speed (∼350 nm/s) occurs when Myo19 is supplemented with CaM, but not RLC12B, suggesting maximal motility requires binding of CaM to IQ-1 and IQ-3. The addition of calcium slowed actin gliding (∼200 nm/s) without an apparent effect on CaM affinity. Furthermore, we show that small ensembles of Myo19 motors attached to quantum dots can undergo processive runs over several microns, and that calcium reduces the attachment frequency and run length of Myo19. Together, our data are consistent with a model where a few single-headed Myo19 molecules attached to a mitochondrion can sustain prolonged motile associations with actin in a CaM- and calcium-dependent manner. Based on these properties, we propose that Myo19 can function in mitochondria transport along actin filaments, tension generation on multiple randomly oriented filaments, and/or pushing against branched actin networks assembled near the membrane surface.

Mitochondrial dynamics are paramount for cell survival and are perturbed in neurodegenerative diseases, hearing loss, and cancer (1, 2, 3). Mitochondrial health requires fusion and fission events that ensure an even inheritance of undamaged proteins and mitochondrial DNA. In addition, mitochondria need to be redistributed appropriately before cell division. Myosin-19 (Myo19) has critical roles in mitochondrial partitioning (4, 5), regulating the balance of fission and fusion (6), and has recently been implicated in cristae organization (7). Myo19 dysregulation is associated with glioma, breast cancer, and hearing impairment (8, 9, 10). How Myo19 participates in these functions at the molecular level remains an open question.

Myo19 belongs to its own class-19 subgroup of the myosin superfamily that shares a conserved, plus end-directed motor domain (MD), a lever arm containing three light chain (LC)–binding IQ motifs, and a unique tail region termed the Myosin Mitochondrial Outer Membrane–Associated (MyMOMA) domain that directs Myo19 to mitochondria (Fi. 1*A*) (11, 12, 13, 14). The MyMOMA domain contains a putative monotopic membrane insertion segment and a segment that interacts with the mitochondrial Rho GTPases, Miro1 and Miro2 (15, 16, 17). Myo19 was recently found to associate with the SAM–MICOS complex that bridges the outer and inner mitochondrial membranes and may thus act as a mechanical tether for cristae formation and regulation (7).

**Figure 1:**
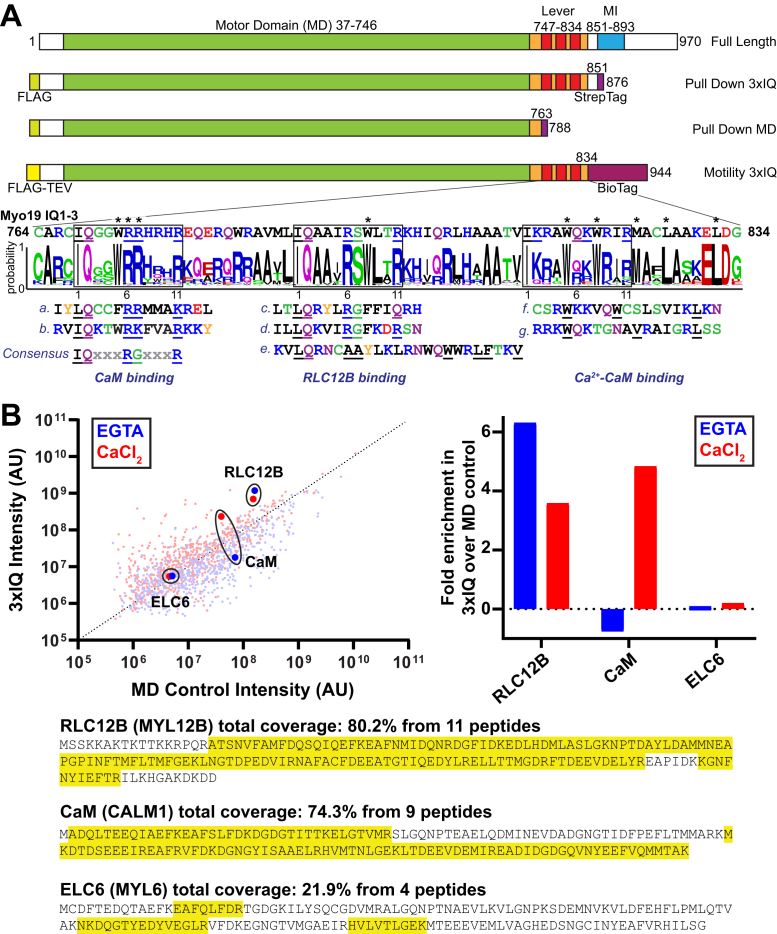
**Identification of LCs by mass spectrometry (MS)–based proteomics.***A*, Myo19 sequences. *Top*, domain diagram of Myo19 and employed fragments. BioTag: biotinylation tag (Experimental procedures section). *Bottom*, consensus sequence of Myo19’s lever arm region aligned to IQ motifs (a–g) that have known LC interactions (below in *italics*) chosen based on sequence similarities with Myo19 (Q96H55). The motifs are (a) Myo5A IQ6 (Q9Y4I1; 860–905), (b) Myo1E IQ1 (Q63356; 700–715), (c) Myo15 IQ1 (Q9QZZ4; 1891–1905), (d) Myo7A IQ1 (Q13402; 749–763), (e) NM-Myo2A IQ2 (P35579; 810–834), (f) Myo6 insert 2 (Q9UM54; 790–808), and (g) MLCK (Q15746; 1742–1760). All sequences are from human except (b) *Rattus norvegicus* and (c) *Mus musculus* corresponding to published work revealing LC-binding properties. *Boxes* predicted IQ sequences. Established motif residues are *underlined*. *Asterisks*: potential motif residues. *B*, LCs identified by pull-down coupled with MS. *Top left*, total proteins identified by MS in the 12 to 30 kDa range plotted by the relative intensities of their constituent peptides in the spectra to demonstrate relative abundance in Myo19-3xIQ *versus* the MD. *Dotted line*, Myo19-3xIQ equals MD. *Top right*, fold enrichment over the MD (control sample) of each identified LC based on the intensities of peptide peaks. *Bottom*, coverage of LC candidates (*yellow highlight*), RLC12B (O14950) and CaM (P0DP23), and low confidence LC, ELC6 (P60660). Experiments were performed in the presence of 1 mM EGTA or CaCl_2_. CaM, calmodulin; MD, motor domain; Myo19, myosin-19.

A gap in our knowledge regarding the molecular mechanism of Myo19 is the identity of the LCs that bind to its lever arm. Most biochemical studies have been performed assuming calmodulin (CaM) binds the three IQs of Myo19 (12, 14, 18). However, one study identified RLC9 (MYL9/MRLC1) and RLC12B (MYL12B/MRLC2), but not CaM, as the endogenous LCs of Myo19 using proteomics and reconstitution experiments (13). In the proteomic screen, recombinant Myo19 was exposed to a kidney extract to identify novel tissue-level binding partners. It is possible, however, that there are tissue-specific differences in the LC composition of Myo19 or that the IQs were not fully occupied. RLC12B is ubiquitously expressed, whereas RLC9 is a more specialized LC isoform expressed in many but not all tissues. Since Myo19 is also ubiquitously expressed (11), it is possible that RLC9 is not always bound. The primary sequences of the putative IQ motifs of Myo19 deviate to varying degrees from the consensus IQ sequence and provide no obvious indication as to the identity of their binding LCs (Fig. 1*A*). For several myosins, the associated LCs were identified by purifying the motors from native tissue (reviewed in Ref. (19)), but this strategy is sometimes problematic if the myosin is not highly expressed at the endogenous level. Alternatively, recent studies have taken the approach of systematically coexpressing combinations of candidate LCs with the heavy chain (HC) to identify sets of LCs that enable robust recombinant expression, solubility, and *in vitro* motor activity of different unconventional myosins (20, 21, 22, 23, 24). These studies show that the LCs can differentially affect motor activity, and the wrong pairing of LCs with the HC can result in incomplete occupancy and protein aggregation. It is known that when the IQ motifs in the lever arm are unoccupied, myosin is unstable and prone to aggregation (25). Therefore, the exact composition of the complete myosin–LC complex is at the core of the motor’s ability to function. To fully understand the functional and regulatory properties of Myo19 *in vitro*, the LC binding partners need to be firmly established.

This study identifies and validates the LCs associated with Myo19 in cultured human cells and elucidates the regulatory effects of these LCs on the motor activity. We find that the second IQ of Myo19 binds RLC12B, whereas IQs 1 and 3 bind CaM. Maximal motility requires the presence of excess CaM, which seems to dissociate from one of the IQs during purification, whereas the tighter-binding RLC12B remains associated. Our results indicate that Myo19 motility is unusually insensitive to calcium, which weakens CaM binding to the lever of other myosins. Ensembles of Myo19 can transport a small cargo over multiple microns both in the presence or in the absence of calcium. Together, these findings reveal emerging sequence determinants for nonclassical IQ binding by distinct LCs. Our work in addition supports a model whereby in the cell Myo19 is bound to both RLC12B and CaM irrespective of calcium levels, allowing the motor to generate actin-based tension near the mitochondrial surface.

## Results

### Myo19 binds RLC12B and CaM

To elucidate the LCs natively bound to Myo19, we expressed a truncated Myo19 construct (amino acids 1–851; Myo19-3xIQ) with dual N-terminal FLAG and C-terminal Streptactin affinity-purification tags (Fig. 1*A*) in a human cell line (Expi293). This truncation was used to overcome difficulties with the expression and purification of full-length Myo19. We identified binding partners of Myo19-3xIQ by mass spectrometry (MS)–based proteomics. Since some LCs bind in a calcium-dependent manner, proteomics experiments were performed in the presence and the in the absence of calcium. Myo19-3xIQ displayed a calcium-insensitive enrichment of MYL12B, hereafter referred to as RLC12B, compared with the MD construct that lacks the IQ motifs (Fig. 1*B*). CaM was also identified, but its enrichment in the Myo19-3xIQ sample over the MD control required the addition of calcium. The cytoplasmic essential LC MYL6 (referred to here as ELC6) was also detected, but its abundance in the spectra was comparable to that of other contaminants, which included other myosins, suggesting lack of specific binding to Myo19. No other LC was detected with sufficient confidence, and specifically MYL9 (RLC9) was not identified, consistent with a lack of RLC9 expression in human embryonic kidney 293 cells (Human Protein Atlas, proteinatlas.org).

**Figure 2:**
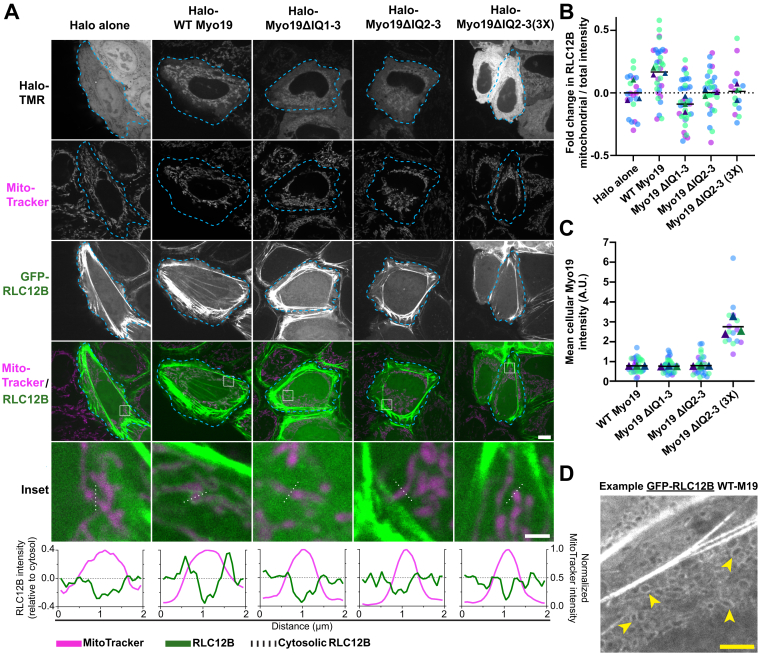
**Full-length Myo19 overexpression recruits GFP-RLC12B to mitochondria.***A*, panels show example cells (*dotted outline*) transfected with Halo-tagged constructs and GFP-RLC12B and stained with MitoTracker Deep Red. *Insets* feature mitochondria where *dotted lines* indicate line scans (*below*). Scale bars represent 10 μm and 2 μm for *insets*. Note: GFP-RLC12B channels are all contrasted the same, whereas the MitoTracker channels are differentially contrasted to normalize the appearance of mitochondria. *B*, fold change in GFP-RLC12B perimitochondrial intensity over total mean intensity normalized to Halo alone. For Myo19 ΔIQ2–3, cells with high Halo-Myo19 expression (3×) show that failure to recruit RLC12B is not because of the number of IQs deleted. One-way ANOVA: for Halo alone *versus* WT Myo19, *p* = 0.0433 (N = 3 trials) or *p* = 0.0009 (n_1_ = 22, n_2_ = 34 cells); Halo alone was not significantly different from ΔIQ1–3, ΔIQ2–3, and 3× ΔIQ2–3 (*p* = 0.42–0.99). *C*, levels of Halo-TMR for cells corresponding to those in *B*. *B* and *C*, *light circles*: individual cells; *dark triangles*: averages of trials. Colors correspond to each trial. *D*, example of WT Myo19-overexpressing cell zoomed in to show GFP-RLC12B outlining mitochondria (*arrowheads*; Fig. S1). This example shows a relatively high level of mitochondrial localization within the dataset and was contrasted differently than the corresponding panels in *A* to clearly show GFP-RLC12B encircling mitochondria. Bar represents 2 μm. Myo19, myosin-19.

To test whether RLC12B interacts with Myo19 in live cells, we expressed Halo-Myo19 and GFP-RLC12B in HeLa cells and used confocal microscopy to determine whether Myo19 overexpression drove the recruitment of RLC12B to mitochondria. GFP-RLC12B localized to the cell cortex under all conditions, consistent with its known association with nonmuscle myosin-II, but mitochondrial recruitment was observed upon overexpression of full-length Myo19 but not in cells expressing Halo-tag only (Fig. 2). Moreover, overexpressed GFP-RLC12B did not colocalize with Myo19 constructs lacking IQ domains 1 to 3 or 2 to 3 (Myo19-ΔIQ1–3 and Myo19-ΔIQ2–3), suggesting that RLC12B recruitment to mitochondria is specifically dependent on its interaction with either or both IQ2 and IQ3 (Fig. 2).

**Figure 3:**
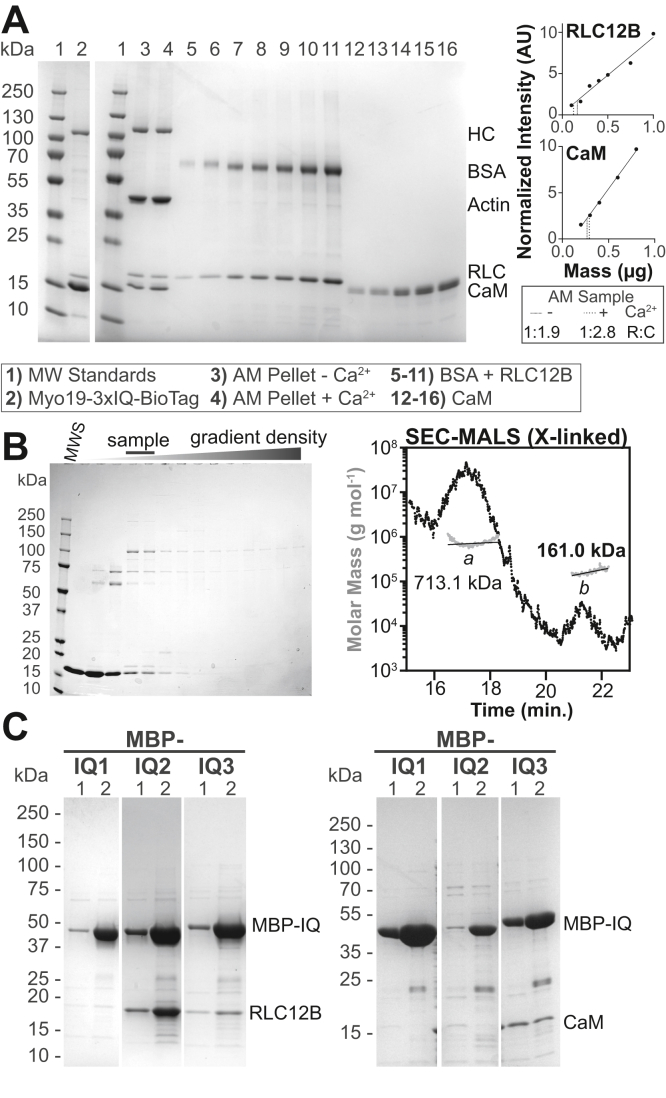
**Copurification of Myo19 with RLC12B and CaM.***A* and *B* (*left*) and (*C*), Coomassie-stained SDS-PAGE gradient gels (4–20%) (*A*) *left*: example preparation of Myo19-3xIQ-BioTag coexpressed with RLC12B and CaM in the *Sf*9/baculovirus system. CaM is added during purification. *Right*, precentrifuged Myo19 was copelleted with actin ± 0.1 mM buffered-free calcium; pellet fractions shown (lanes 3–4) as well as standards (lanes 5–16) used to generate curves (*graphs*). *B*, *left*, gel of glycerol gradient fractions of unfixed samples. *Left to right*, samples from the fractions (*gradient triangle*; lanes 2–15) were run on the gel from low to high glycerol with fractions shown: 4, 7, 9, 12, 13, 15, 17, 19, 21, 23, 25, 27, 29 and the *bottom* of the tube. *Right*, GraFix fractions corresponding to the fractions labeled “sample” shown on the gel were analyzed by SEC–MALS. *C*, maltose-binding protein (MBP)-IQ-6xHis fusion proteins were coexpressed with (*left*) RLC12B or (*right*) CaM and purified using nickel chromatography (full gels with purification steps shown in Fig. S2). Lane 1: 30 mM imidazole wash and lane 2: 250 mM imidazole peak elution fraction. See Experimental procedures section for IQ sequences. AM, acto-Myo19; BSA, bovine serum albumin; CaM, calmodulin; HC, heavy chain; MALS, multiangle light scattering; MW, molecular weight; Myo19, myosin-19; SEC, size-exclusion chromatography.

Based on the aforementioned evidence, we elected to reconstitute Myo19 with RLC12B and CaM by coexpression and purification using the Sf9-baculovirus system. Consistent with the finding in human cells, Myo19-3xIQ copurified with both RLC12B and CaM (Fig. 3*A*). Since CaM generally disassociates from myosins during purification (see the Experimental procedures section), excess CaM was added, resulting in excess CaM in the gel. To estimate the stoichiometry of the LCs bound to the motor, this Myo19-3xIQ preparation was cosedimented with actin filaments and evaluated by gel densitometry. The CaM to RLC12B ratio was determined to be 1.9:1 in the absence of calcium using known concentrations of these proteins as densitometry standards (Fig. 3*A*). The ratios of LCs to HC were 1.8 RLC12B and 3.4 CaM when bovine serum albumin was used to determine HC concentration. While the RLC12B to CaM ratio determined in this manner appears approximately accurate, the LC:HC ratios are clearly inaccurate as the motor contains three IQ motifs. Incorrect ratios are possibly because of differential dye binding to the LCs, the HC, and standards. Calcium (0.1 mM free) resulted in a CaM:RLC12B ratio of 2.8:1, suggesting that the affinity of CaM-Myo19 is not weakened upon CaM–Ca^2+^ binding. This finding was consistent with our MS result where CaM was detected predominantly in the presence of calcium.

Given the difficulties in determining the LC to HC ratios, glycerol gradient centrifugation (without calcium) was used as an alternative way to resolve the motor complex from free LCs (Fig. 3*B*). The complex was stabilized by fixation during gradient centrifugation (GraFix) (26) to obtain stable samples for mass determination. We determined mass of the crosslinked complex by size-exclusion chromatography combined with multiangle light scattering, which showed a main peak of 161.0 ± 1.5 kDa that approximately matches the expected molecular weight of a 1:2:1 HC–CaM–RLC12B complex (159.6 kDa), and a peak of 713.1 ± 7.1 kDa, which was the result of intermolecular crosslinks (Fig. 3*B*). Combined, our data suggest that the 3-IQ motifs of Myo19 bind CaM and RLC12B at a 2:1 ratio.

To determine which of the three IQ motifs binds RLC12B, we assayed the ability of RLC12B and CaM to copurify with maltose-binding protein (MBP)-IQ-6xHis fusion proteins, each containing one of the three IQs. When coexpressed in *Escherichia coli*, RLC12B copurified stoichiometrically with IQ2, which showed no interaction with CaM (Fig. 3*C*). IQ1 did not copurify with any candidate, and IQ3 copurified with substoichiometric amounts of both, indicating that IQ1 and IQ3 are less specific and lower-affinity sites prone to LC dissociation. It is also likely that the isolated IQs have altered properties when outside the context of the motor, and therefore, we proceeded to address the effects of the different LCs on motor activity.

### Maximal actin gliding by Myo19 requires excess CaM

**Figure 4:**
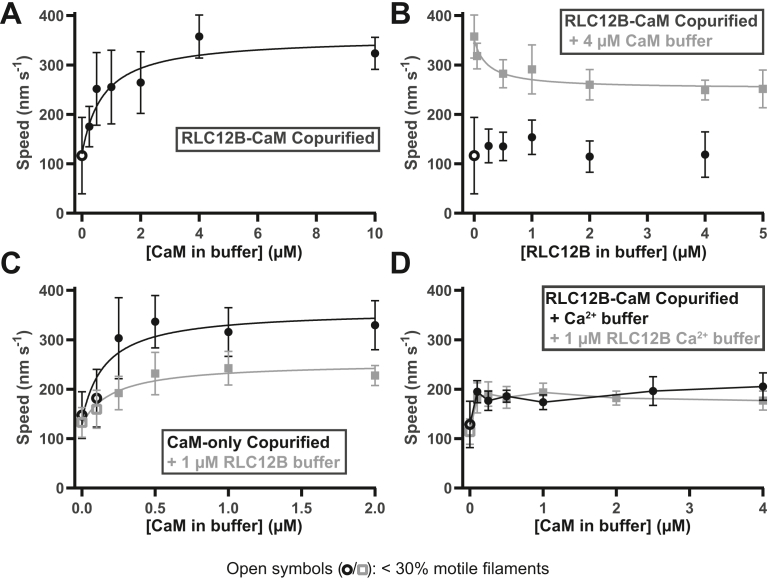
**Gliding velocity is stimulated by excess CaM.***A*, *B*, and *D*, Myo19-3xIQ-BioTag copurified with RLC12B and CaM or (*C*) Myo19 purified without RLC12B coexpression was employed in gliding motility assays (see the Experimental procedures section). Concentrations show what CaM and/or RLC12B was added in the buffer, not accounting for what was introduced by the myosin preparation. *D*, the experiment was performed with 0.1 mM free calcium (see the Experimental procedures section) added to the buffer along with indicated LC(s). *A*–*C*, *solid lines*: hyperbolic fits; (*D*) *solid lines*: connect the points. Error represents SD. Data in *A* and *B* are combined from five trials using N = 2 preparations of Myo19. Points: mean speeds of n = 40 filaments except: no LC addition (n = 34), 2 μM CaM (n = 100), 4 μM CaM (n = 60), 10 μM CaM (n = 20), and 2 μM RLC12B (n = 140). Data in *C* and *D* each represent their own preparation of Myo19 (N = 1); points: n = 20 filaments. CaM, calmodulin; Myo19, myosin-19.

Given the apparently weak affinity of CaM for at least one of the IQ motifs, we next examined the effect of supplementing Myo19 with CaM, RLC12B, or both in actin-gliding motility assays *in vitro*. For these experiments, we employed Myo19-3xIQ-BioTag copurified with RLC12B and CaM through FLAG-affinity and ion exchange chromatography. The BioTag moiety used for attaching Myo19 with streptavidin to surfaces (coverslips and quantum dots [Qdots]) ensures that the protein is efficiently biotinylated during expression, eliminating the need for additional biotinylation steps *in vitro* (27). Motility experiments were performed at 37 °C to more closely model *in vivo* conditions. Because of excess CaM present in the Myo19 preparations (Fig. 3*A*), obtaining accurate concentrations of the complex by Bradford assay was problematic. We determined that 40 μg/mL of these preparations applied to motility chambers was sufficient to drive maximal motility under optimal conditions, which we used in all the experiments (described later). When no excess LC was added, filament sliding proceeded with an average speed of 120 ± 80 nm s^−1^ (SD), and most (>70%) filaments were nonmotile (static or undergoing reptation; Figure 4*A*). The addition of CaM made the motility more consistent across the field of view with constant, uniform motion, and the gliding speed increased to 360 ± 40 nm s^−1^ with 4 μM CaM added (*p* < 0.0001 *versus* no LC; Fig. 4*A*). A hyperbolic fit of the data yielded an EC_50_ of 0.7 ± 0.6 μM (SE) and a plateau of 360 ± 50 nm s^−1^ (SE). The addition of RLC12B also promoted uniform motility but only marginally increased the speed. The maximal speed attained with 1 μM excess RLC12B added, 150 ± 30 nm s^−1^, was significantly lower than the maximal speed with 4 μM excess CaM (*p* < 0.0001; Fig. 4*B*). When RLC12B (1 μM) and CaM (4 μM) were added together, the gliding velocity was reduced compared with the maximal velocity observed with excess CaM (4 μM) alone (*p* < 0.0001; Fig. 4*B*). With excess CaM present at a fixed concentration (4 μM), increasing RLC12B concentrations resulted in a downward trend, which we fit to an inhibition model yielding an IC_50_ of 0.2 ± 0.3 μM and a plateau of 250 ± 20 nm s^−1^ (Fig. 4*B*). This result suggests partial competition of RLC12B with CaM for binding to the low-affinity IQs. We interpret these data to mean that CaM, not RLC12B, occupies the two low-affinity IQs surrounding the high-affinity RLC12B-binding IQ2 to allow maximal motility of Myo19.

We next asked whether Myo19 must specifically have RLC12B bound to IQ2 for maximal sliding velocity or whether CaM binding to all three IQs would enable similar velocities. To address this question, Myo19 was coexpressed with CaM alone and purified as before, so that only CaM was available to bind. Gliding motility assays were then performed under a range of CaM concentrations, with or without 1 μM RLC12B added. When CaM was added alone, the sliding velocity increased to a maximum of 340 ± 50 nm s^−1^ at 0.5 μM CaM (fit plateau: 360 ± 40 nm s^−1^; EC_50_: 0.2 ± 0.1 μM). In the presence of 1 μM RLC12B, the maximum speed was 240 ± 30 nm s^−1^ at 1.0 μM CaM (fit plateau: 250 ± 20 nm s^−1^; EC_50_ = 0.2 ± 0.1 μM; Fig. 4*C*). When comparing Myo19 copurified with both RLC12B and CaM (Fig. 4*A*) with Myo19 copurified with CaM only (Fig. 4*C*), both reach a maximum speed of approximately 350 nm s^−1^ when only excess CaM is added (*p* = 0.1245, comparing maximum rates of each), suggesting that CaM can also bind IQ2. There was a consistent reduction in speed (to ∼250 nm s^−1^) when 1 μM RLC12B was added in solution in the presence of CaM (Fig. 4, *B* and *C*), which is potentially because of excess RLC12B binding ectopically to IQ1 and/or IQ3. Therefore, while both CaM and RLC12B can bind and stabilize IQ2 and have similar effects on the sliding velocity, RLC12B binds this IQ with higher affinity and outcompetes CaM when coexpressed, which more closely mirrors physiological conditions where both LCs are present.

### Calcium reduces the speed, run length, and filament-binding frequency of Myo19

Calcium is a strong inhibitor of CaM binding to some unconventional myosins (28, 29, 30, 31). To examine whether this mechanism also applies to Myo19–CaM interactions, gliding motility assays were performed in the presence of 0.1 mM free calcium over a range of CaM concentrations and in the presence or the absence of 1 μM excess RLC12B. For these assays, we resumed using Myo19-3xIQ copurified with both RLC12B and CaM to best approximate the native motor. Under these conditions, 0.1 μM additional CaM was sufficient to increase the speed from a baseline rate of 130 ± 10 nm s^−1^ to 190 ± 20 nm s^−1^ (*p* < 0.0001), close to the maximum speed at 4 μM CaM–Ca^2+^ (210 ± 30 nm s^−1^; *p* = 0.1909; Fig. 4*D*). Rates with 1 μM additional RLC12B (180 ± 30 nm s^−1^ for 0.1 μM CaM) were nearly identical to when only CaM was added (*p* = 0.1468). Strikingly, CaM–Ca^2+^ promotes the same rate, ∼200 nm s^−1^, over a concentration range of two orders of magnitude (10 μM CaM–Ca^2+^: 180 ± 20 nm s^−1^; Fig. 4*D*), suggesting that the reduced velocity compared with without calcium (4 μM CaM–Ca^2+^ *versus* 2 μM CaM control: 390 ± 90 nm s^−1^; *p* < 0.0001) is not an effect of weakened Myo19–CaM affinity but rather an allosteric effect on the motor.

**Figure 5:**
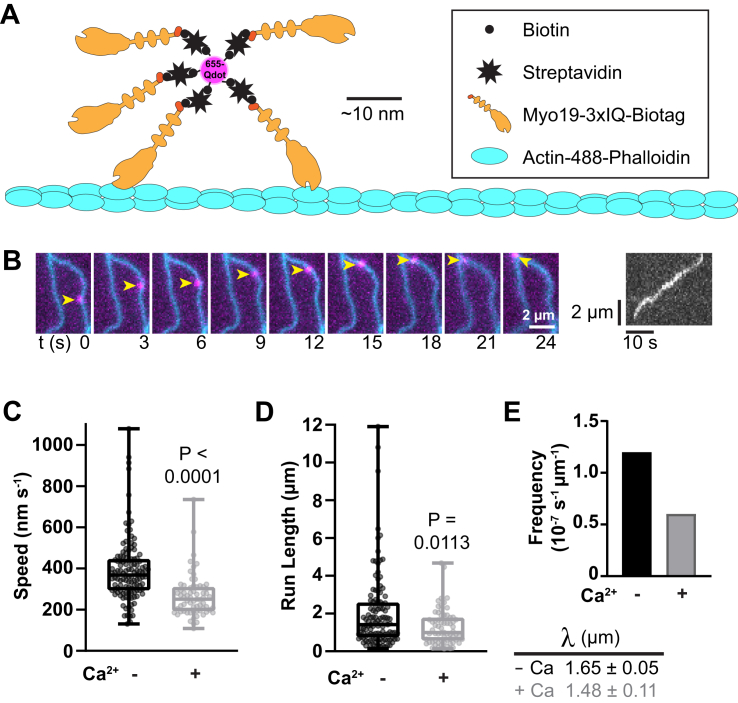
**Qdot transport by Myo19 ensembles is negatively modulated by calcium.***A*, diagram of Qdot-transport assays (see the Experimental procedures section). *B*, example time series and kymograph of a Qdot-Myo19 ensemble (*magenta*) moving along an actin filament (*cyan*). Effect of 0.1 mM free calcium on (*C*) speeds, (*D*) run lengths, and (*E*) run frequencies (events over time and length of actin) of Qdot-Myo19 ensembles. Run length data fit to single exponentials yielded run length constants λ (see the Experimental procedures section). Statistics: (*C*) Welch’s *t* test; (*D*) Mann–Whitney test. Data in *C* and *D* are from three trials employing two independent preparations of Myo19 (N = 2). We noted a general trend during each trial that there were consistently more runs in the calcium-free condition (total n = 117) than with calcium (n = 67). For optimal comparison, frequencies (*E*) were shown from a single trial that had the overall highest frequencies representative of both conditions. Myo19, myosin-19; Qdot, quantum dot.

The Qdot-transport assay is sensitive to perturbations of myosin processivity and actomyosin affinity (Fig. 5*A*). To further explore why calcium reduces the velocity of Myo19, Qdots bound to ensembles of Myo19-3xIQ were assayed for their ability to undergo processive runs with or without 0.1 mM free calcium. Under calcium-free conditions, Myo19 ensembles performed multimicron processive runs, sometimes limited to the lengths of the actin filaments (Fig. 5*B*). Qdot speeds, 390 ± 160 nm s^−1^, were consistent with those observed in the gliding assay and decreased proportionally when calcium was added (270 ± 100 nm s^−1^; Fig. 5*C*). Qdot run length also decreased with calcium (Fig. 5*D*). Strikingly, in the presence of calcium, the frequency of processive runs was reduced 50% compared with the calcium-free condition (Fig. 5*E*). Together, these data show that Myo19 ensembles decorated with either CaM or CaM–Ca^2+^ support processive motility and that calcium reduces the speed, run length, and frequency of motile events, likely because of a decrease in actomyosin affinity.

## Discussion

Here, we uncover the differential roles of RLC12B and CaM in regulating Myo19 motility, establish that calcium modulates actin gliding and cargo transport, and show that ensembles of single-headed Myo19 motors can transport Qdots. These findings have important implications for the molecular mechanisms underlying the cellular functions of Myo19 in facilitating mitochondrial dynamics and morphogenesis. Together, our results shed light on what constitutes a minimal functional Myo19 motor, complete with one RLC12B and two CaMs.

The binding of RLC9–RLC12B or CaM was shown by previous studies (12, 13, 14), but we find that, among these LCs, CaM stimulates maximal velocities (Fig. 4), consistent with Myo19 interacting with CaM in a conformational state that best stabilizes the lever arm. The difference in speed of Myo19 decorated with RLC12B or CaM only, ∼150 *versus* ∼350 nm s^−1^, was remarkably similar to published work by other groups when comparing the rate of ∼50 nm s^−1^ when only RLC9–RLC12B was bound (13) and ∼230 nm s^−1^ when only CaM was bound (12), accounting for the temperature differences (the other studies used ∼25 °C, whereas our experiments were done at 37 °C). Our *in vitro* rates are also similar to speeds of Myo19 puncta moving along filopodia in live cells (∼200–400 nm s^−1^) (32). RLC12B strongly interacts with IQ2 of Myo19 as shown by proteomics, cellular colocalization, and copurification (Figure 1, Figure 2, Figure 3) but is not required for maximal gliding velocity in motility assays (Fig. 4*C*). Since the working stroke of myosin propagates first from the converter through IQ1 and then toward the rest of the lever, it is likely that CaM specifically binding to IQ1 is critical for establishing a rigid interface between the motor and the lever arm. In contrast, IQ2–3 may be less susceptible to the specific nature of the LCs that stabilize the lever arm.

CaM shifts conformation upon calcium binding and opens up the N- and C-terminal lobes, exposing hydrophobic side chains to the solvent (33). This shift causes CaM to dissociate from some previously characterized myosins’ IQs (including myosins of classes 1, 5, and 6) and bind numerous nonmyosin targets in the cell (28, 29, 30, 31, 34). Unlike these myosins, Myo19 can sustain motility in high calcium conditions at an intermediate velocity (∼200 nm s^−1^), even when the CaM concentration is submicromolar (Figs. 4 and 5). This evidence suggests that CaM–Ca^2+^ does not dissociate from Myo19 but rather stays bound while adopting the open conformation. The open conformation of CaM on IQ1 is expected to change the interface with the converter domain, which would likely affect the motor stepping. In support of this notion, calcium was found to decrease the number and length of association events of Myo19 ensembles on Qdots (Fig. 5, *C* and *D*). The converter domain of Myo19 is unique in that it contains a large 38-amino acid insert (residues 680–717), which, unlike the insert in myosin-6, does not cause a change in motor directionality (13). The role of this insert is unknown, but the converter is located directly at the interface that may affect the communication between the motor and the lever (35). For example, an insert in the converter of myosin–IB interacts directly with the CaM bound to IQ1, positioning the lever arm helix at a unique angle relative to the motor (36, 37). Thus, the unique insert of Myo19 may play a role in reorienting the lever arm helix or possibly conveying the calcium state of CaM to the MD. Structural studies will be required to elucidate the underlying mechanism of calcium’s unique modulatory effect on Myo19–CaM. The cellular relevance of Myo19’s modulation by calcium is undetermined, yet mitochondria are key regulators of cytoplasmic calcium flux (38) and it could be advantageous for Myo19 to have weak calcium sensitivity.

IQ motifs have both specificity and adaptability in binding different LCs. The rules governing specificity remain undefined with few exceptions. For example, for class-II myosins, RLC binding is partially defined by an IQ motif that has a unique WxW motif marking a ∼90° bend in the lever arm α-helix with the two lobes of the RLC binding on opposite sites from this bend (39). Recently, however, Myo7A and Myo15 have been identified as targets of RLC12B, which binds tightly to IQ1 of these myosins (21, 22, 24). These IQs, similar to IQ2 of Myo19, do not have the WxW motif associated with RLC binding and are likely specific for RLC12B binding through still-to-be-identified interactions (Fig. 1*A*). These unconventional RLC12B-targeting sites generally follow the typical IQ consensus sequence (IQxxxRGxxxR), and it is unclear what sets them apart from other IQs that bind CaM preferentially. The residues in position 8 have an aromatic side chain (W or F) in these three examples, but this is not uncommon among other IQs as well (Fig. 1*A*) (40). It is likely that the N-terminal lobe of the RLC12B, which normally binds the WxxLFxxV motif in class-II myosins, recognizes sequences outside the conventional IQ motif.

The CaM-binding sites of Myo19, IQs 1 and 3, are even less conforming to the consensus IQ sequence than IQ2. IQ1 has an R instead of G at position 7, but examples of CaM-binding IQs that have R or K in this position can be found in other myosins, including Myo5A (IQ6) and Myo1E (IQ1) (Fig. 1*A*). Notably, Myo1E remains bound to CaM in the presence of calcium albeit its ATPase activity is reduced (41). Myo1E’s single IQ and the first IQ of Myo19 appear to have common features (IQxxWR[R/K]xxxR) as well as the shared ability to bind Ca^2+^–CaM. The steric clash from Arg at position 7 is also predicted to cause the N-terminal lobe of CaM to project outward rather than bind the IQ (42). Of the three IQs of Myo19, IQ3 is the most divergent, with K residue instead of Q at position 2 and QK instead of RG at positions 6 to 7 (Fig. 1*A*). The absence of key consensus IQ residues and the highly conserved Trp residue in position 5 are properties shared with the insert-2 sequence of Myo6, which functions as the first CaM-binding site adjacent to the converter, and the Ca^2+^–CaM-binding site of myosin LC kinase (Fig. 1*A*). Interestingly, the structure of Myo6 suggests that insert-2 binds CaM in an unusual contracted-open conformation that does not allow calcium to dissociate (43). Based on Myo19’s sequence and Ca^2+^–CaM-binding properties, we postulate that IQ3 is not a conventional IQ motif and is more closely related to the insert-2 of Myo6.

Myo19 has been proposed to be a processive high duty ratio motor with the ability to transport mitochondria in the cell (18, 32, 44). Kinetics studies provide strong evidence that Myo19 has a relatively high duty ratio, which would be conducive to processivity (14, 18). Supporting evidence of processivity came from a study where Myo19 artificially dimerized with a leucine zipper exhibited short and processive movements of 0.18 μm and up to ∼0.3 μm maximum displacement (32). Although this evidence points to Myo19 potentially functioning as a transporter motor, there is no clear evidence showing that Myo19 functions as a dimeric motor endogenously. The tail domain sequence shows neither predicted coiled-coil or other dimerization motif that would permit self-interaction nor has any binding partner been shown to dimerize Myo19. Thus far, the only known role of the tail domain is recruiting Myo19 to the mitochondrial outer membrane (11, 15, 17). Assuming that multiple single-headed Myo19 molecules bound to a single mitochondrion could produce processive movements, we proceeded to reconstitute a biomimetic system using Qdots saturated with Myo19 motors lacking the tail domain. Under these conditions, Myo19 ensembles were highly processive with a typical run length of 1.6 μm and up to nearly 12 μm (Fig. 5), substantially longer than artificially dimerized Myo19 (32). The longer run lengths exhibited in our reconstituted system are consistent with more physiological-scale distances, that is, microns-long filopodia, and an overall higher efficiency of motion. Therefore, it is plausible that Myo19 could function processively as a collection of motors bound to a mitochondrion, yet our data cannot exclude possible mechanisms involving dimerization through unknown interactions. Moreover, high duty ratio motors that are capable of processive transport may also be suitable for anchoring functions that stably link associated organelles with actin filaments. The behavior of mitochondria mediated by Myo19 could range from highly dynamic to completely static, depending on the valency of actomyosin interactions and the relative origin and orientation of the actin filaments. Understanding when and where Myo19 is active in the cell will be critical for understanding how Myo19 functions.

While our data show that the Myo19 motor has properties that enable ensembles to transport Qdots *in vitro*, the precise role of Myo19 motor activity in the cell is poorly understood. Myo19-mediated directed motions have been shown to occur along filopodia under specific conditions, when Myo19 is overexpressed and cells undergo glucose starvation (18, 32, 44). Under growth conditions, Myo19 activity is implicated in tethering mitochondria to dynamic actin networks to evenly disperse mitochondria in the cytoplasm before cell division (4, 5, 6). During dispersion, mitochondria undergo different dynamics depending on the associated actin structure; actin clouds and cables produce submicron displacements, whereas comet tails can move mitochondria several microns (4). Based on our current findings, Myo19 ensembles at the mitochondrion surface should be able to associate with these different actin structures and apply barbed end-directed force for several seconds without dissociating. This behavior may be advantageous for dynamic anchoring and allowing mitochondria to associate processively toward the plus (growing) end of some of these mitochondrial-associated dynamic actin structures. Myo19 is also involved in mitochondrial fission, which is preceded by mitochondria preconstriction at the fission site depending on randomly oriented actin filaments surrounding mitochondria (6, 45). A plausible role of Myo19 ensemble processivity is to contribute to preconstriction by pulling a mitochondrion in different directions simultaneously along these multidirectional actin networks. The ability of Myo19 to induce mitochondrial preconstriction remains to be directly examined. Finally, other roles for Myo19 may involve mitochondrial–endoplasmic reticulum and/or mitochondrial–lysosomal contacts (46, 47), for which an understanding of its motor activity and regulation will provide deep insight.

## Experimental procedures

### Sequence analysis

The consensus sequence of the lever arm of Myo19 was created by aligning full-length sequences of Myo19 from 55 organisms spanning vertebrata from humans to channel catfish (*Ictalurus punctatus*). Sequence IDs (amino acids in parentheses) were Q96H55 (764–834), Q5SV80 (764–834), A0A6P5QGB4 (764–834), A0A6J0DWE2 (761–831), I3ML92 (764–834), H0V2S3 (757–827), A0A1S3EZK3 (764–834), H2QCR4 (764–834), A0A2R9AXU1 (764–834), G3QY42 (764–834), A0A2K5WEM0 (763–833), A0A5F7ZMQ5 (784–854), A0A096P509 (764–834), A0A2K5NMX7 (764–834), A0A2K5IBB4 (764–834), A0A2K5DPV4 (764–834), F7BED0 (764–834), A0A2K6ELK8 (758–828), G3T3X5 (766–836), A0A6P4U2Y4 (783–853), A0A6J1XC86 (783–853), M3WFE8 (764–834), A0A6I9IYC4 (770–840), A0A384CFN0 (785–855), A0A3Q7WJX5 (796–866), A0A452SCT8 (764–834), G1LNM4 (764–834), A0A2Y9I8J8 (763–833), A0A3Q7NUK0 (763–833), A0A3Q7SA51 (760–830), G3MZU4 (747–817), A0A6P5DIZ3 (747–817), A0A6P7EIM7 (747–817), A0A452G219 (747–817), A0A2Y9LFM9 (758–828), A0A2U4C652 (758–828), A0A6J0AY11 (760–830), G1PM65 (764–834), A0A6P3RBN3 (760–830), A0A6J2MD46 (760–830), F7CK88 (765–835), A0A1S3W3P6 (760–830), G1SEC2 (759–829), A0A6P5KPB5 (765–835), A0A3Q2TZU2 (765–835), 0A1V4K263 (765–835), H0ZCE2 (765–834), A0A6I9HN03 (765–835), A0A6J0HRI7 (765–835), A0A091IJ51 (774–844), A0A6P7P858 (757–827), A0A6J2R2M5 (766–836), H2M6S2 (755–825), A0A1S3RN45 (766–836), and A0A2D0QDW7 (774–844). The sequence logo (Fig. 1*A*) was generated using online WebLOGO software (weblogo.berkeley.edu).

### Pulldowns of Expi293 expressed Myo19 fragments and MS (LC–MS/MS)

The Expi293 Expression System Kit (Thermo Fisher Scientific) was employed according to the manufacturer’s protocol to express different-length Myo19 fragments fused N-terminally to a FLAG tag and C-terminally to a Strep-Tactin tag (WSHPQFEK; Fig. 1*A*). Cells were suspended and lysed in lysis A buffer (50 mM Hepes [pH 7.5], 150 mM NaCl, 5 mM MgCl_2_, and 1 mM EGTA) with 0.5% IGEPAL ca-630, 25 mM sucrose, cOmplete Mini Protease inhibitor cocktail (catalog no.: 11836170001; Roche), 1 mg mL^−1^ benzamidine, 1 mM PMSF, and 5 mM ATP by 40 strokes with a dounce homogenizer on ice. Lysates were clarified at 53,000*g* for 30 min at 4 °C, and proteins were first bound by Strep-Tactin XT resin (IBA Lifesciences), washed with 100 column volumes (CVs) of lysis A, eluted with Strep Elution buffer (50 mM biotin, 250 mM Hepes [pH 7.5], 150 mM NaCl, and 1 mM EGTA), bound to anti-FLAG affinity resin, washed with 50 CV lysis A, and then eluted with FLAG Elution buffer (0.5 mg mL^−1^ 3xFLAG peptide, 20 mM Hepes [pH 7.5], and 100 mM NaCl). Peak fractions were pooled and concentrated using centrifugal filtration units, and then samples were immediately boiled in SDS loading dye. These assays were also performed with 1 mM CaCl_2_ added instead of EGTA.

LC–MS/MS analysis was performed by the Proteomics Facility at The Wistar Institute using a Q Exactive HF mass spectrometer (Thermo Fisher Scientific) coupled with a Nano-ACQUITY UPLC system (Waters). Samples were digested in-gel with trypsin and injected onto a UPLC Symmetry trap column (180 μm i.d. × 2 cm packed with 5 μm C18 resin; Waters). Tryptic peptides were separated by reversed-phase HPLC on a BEH C18 nanocapillary analytical column (75 μm i.d. × 25 cm, 1.7 μm particle size; Waters) using a 90 min gradient formed by solvent A (0.1% formic acid in water) and solvent B (0.1% formic acid in acetonitrile). Eluted peptides were analyzed by the mass spectrometer set to repetitively scan *m/z* from 400 to 2000 in positive ion mode. The full MS scan was collected at 60,000 resolution followed by data-dependent MS/MS scans at 15,000 resolution on the 20 most abundant ions exceeding a minimum threshold of 20,000. Peptide match was set as preferred, exclude isotopes option, and charge-state screening were enabled to reject singly and unassigned charged ions. Peptide sequences were identified using MaxQuant 1.6.17.0 (Max Planck Institute of Biochemistry; 48). MS/MS spectra were searched against a SwissProt human protein database (6/4/2021) using full tryptic specificity with up to two missed cleavages, static carbamidomethylation of Cys, and variable oxidation of Met and protein N-terminal acetylation. Consensus identification lists were generated with false discovery rates of 1% at protein and peptide levels.

### Live cell analysis

HeLa-M cells (A. Peden, Cambridge Institute for Medical Research) were grown in a 37 °C, 5% CO_2_ incubator in Dulbecco's modified Eagle's medium (Corning; catalog no.: 10-017-CV) supplemented with 10% fetal bovine serum and 1% GlutaMax (Gibco; catalog no.: 35050061). Cells were passaged using trypsin, and before imaging, cells were plated on #1.5 glass-bottom dishes. Transfections and labeling were performed using commercial protocols. Briefly, plasmids were diluted in OPTI-MEM (Gibco; catalog no.: 31985-070), such that the volume was 5% that of the volume of maintenance media, and combined with FuGENE 6 transfection reagent (Promega; catalog no.: E269A) for a 10 min, room temperature incubation. This mixture was added to cells for expression over 48 h. Next, TMR halo ligand (Promega; catalog no.: G825A) was added to cells at approximately 1:2500 dilution for 15 min, followed by two washes. Then, cells were incubated in maintenance media with MitoTracker Deep Red FM at 1:6000 dilution for 10 min and washed once. Finally, cells were put in imaging media, composed of 10% fetal bovine serum and 1% GlutaMAX in Leibovitz’s (Gibco; catalog no.: 11415-064), and set for 30 min in the 37 °C microscopy chamber.

Confocal microscopy was conducted using an UltraView VoX PerkinElmer spinning-disk system on a Nikon Eclipse Ti Microscope with an Apochromat 100× 1.49 numerical aperture oil immersion objective (Nikon) and an EM-charge-coupled device camera (C9100; Hamamatsu Photonics). Microscope acquisition parameters were kept constant, and cells were imaged at random. Since the Halo-alone construct expressed highly, care was taken to only image the dimmer cells so that bleed-through into the RLC channel appeared negligible. Using ImageJ (National Institutes of Health), binary masks generated from the MitoTracker images were used to isolate mitochondrial regions of interest in the corresponding GFP-RLC12B images for intensity analysis. For each cell, mitochondria were segmented using the same intensity threshold, and three values were recorded: mean mitochondrial RLC intensity, mean cellular RLC intensity, and mean Myo19 intensity after background subtraction. Unbiased filtering of the dataset was performed to ensure only cells expressing relatively equal amounts of Myo19 and RLC were compared. Only cells with mean cellular RLC intensities within an arbitrarily determined window spanning half an order of magnitude were included in further analysis. Moreover, for that subset of cells, the highest and lowest Myo19 expressors were excluded in a random manner such that between groups the averages of mean cellular Myo19 intensities was similar.

### Expression and purification of Myo19-3xIQ and RLC

Myo19 constructs (Fig. 1*A*), based on the canonical human isoform (Q96H55-1), containing an N-terminal FLAG tag (DYKDDDDK), the MD, the LC-binding IQ region, and a C-terminal biotin tag (27), were expressed and purified with CaM and RLC12B (O14950-1) as indicated using methods established previously for class-I myosins (49, 50, 51).

RLC12B with an N-terminal MBP-tobacco etch virus (TEV) protease cleavage site moiety was expressed in Rosetta(DE3) cells, which were grown in Terrific broth with antibiotic selection to an absorbance of 1.5 and induced overnight with 1 mM IPTG at 18 °C. Cells harvested by centrifugation (4000*g* for 20 min at 4 °C) were resuspended in ice-cold lysis B buffer (20 mM Tris–HCl [Ph 8], 200 mM NaCl, 1 mM EDTA, 1 mM DTT, 10 μM leupeptin, 1 mM PMSF, and 0.4 mg mL^−1^ benzamidine) and lysed using a microfluidizer (Microfluidics). Lysates were clarified by centrifugation at 48,000*g* at 4 °C for 20 min. Proteins were bound to amylose resin, washed, and RLC was eluted by overnight cleavage with TEV protease (4 °C) and dialyzed for 4 h into Buffer-N (20 mM Tris–HCl [pH 8], 200 mM NaCl, and 10 mM imidazole). The protease was removed by binding nickel resin, and the unbound RLC was then dialyzed into Storage Buffer R (20 mM Tris–HCl [pH 8], 100 mM NaCl, 1 mM EGTA, 50% glycerol, and 2 mM DTT) and stored at −20 °C.

### Cosedimentation assay

To remove aggregates, Myo19-3xIQ-BioTag was thawed from frozen aliquot and centrifuged at 200,000*g* for 10 min. Supernatant was used to make reaction mixtures containing approximately 2 μM Myo19, 4 μM F-actin, and 10 μM CaM in S-Buffer (15 mM Hepes [pH 7.5], 7 mM imidazole [pH 7.5], 125 mM NaCl, 5 mM MgCl_2_, 1 mM EGTA, and 1 mM DTT). To test the effect of calcium, 1.1 mM CaCl_2_ was added (for 0.1 mM free Ca^2+^). Mixtures were centrifuged at 200,000*g* for 20 min, and the pellet fractions were run on SDS-PAGE. The ratio of CaM:RLC12B was determined by gel densitometry using purified bovine serum albumin, RLC12B, and CaM standards.

### GraFix and size-exclusion chromatography–MALS

Glycerol gradient centrifugation with (GraFix) or without fixation was performed similarly to Kastner *et al.* (52). Briefly, proteins were sedimented at 40,000 RPM for ∼16 h in a 5 to 30% glycerol gradient at 4 °C using a Beckman SW 60 Ti rotor. Gradients were prepared in 20 mM Hepes (pH 7.5), 0.2 M NaCl, 5 mM MgCl_2_, and 1 mM EGTA using a Gradient Master device (BioComp Instruments). For experiments with crosslinking, 0.125% (v/v) glutaraldehyde was added to the 30% glycerol solution before preparing the gradient. Crosslinking was quenched by the addition of 40 mM glycine–HCl (pH 7.5). A Piston Gradient Fractionator (BioComp Instruments) was used for fractionation.

Glycerol in samples was removed by quick dialysis, followed by centrifugal concentration. For mass determination, samples were injected into a TSKgel SuperSW2000 column (Tosoh Corporation) before entering a DAWN HELEOS MALS detector and an Optilab rEX refractive index detector (Wyatt Technology Corporation). The Astra software (Wyatt Technology Corporation) was used to calculate molecular masses.

### *In vitro* motility assays

Chambers were assembled between slide and coverslip using double-sided tape and silicon vacuum grease. Coverslips were either coated with 0.5% nitrocellulose (Electron Microscopy Sciences) for actin filament–gliding assays or uncoated for Qdot-transport assays. For filament gliding, chambers were treated with 0.1 mg mL^−1^ streptavidin, 2 mg mL^−1^ casein, 40 μg mL^−1^ biotinylated Myo19, and then exchanged with 100 μL reaction mixtures that contained 10 nM rhodamine–phalloidin labeled actin filaments and LCs (CaM or RLC) as indicated in M-Buffer (10 mM imidazole [pH 7.5], 50 mM NaCl, 5 mM MgCl_2_, and 1 mM EGTA) with 0.5% methylcellulose and M-Additives (2 mM MgATP, 20 mM DTT, 5 mg mL^−1^ glucose, 0.2 mg mL^−1^ glucose oxidase, and 40 μg mL^−1^ catalase). Chambers were then sealed with silicon grease and mounted on the microscope where they were equilibrated for 2 min at 37 °C using an objective heater before time-lapse acquisition. For Qdot assays, biotinylated Myo19 was mixed in 35-fold molar excess of streptavidin-coated Qdots (655 nm) to ensure that most Qdots were bound to small ensembles of myosin heads. Chambers were treated with 0.1 mg mL^−1^ *N*-ethylmaleimide myosin, 2 mg mL^−1^ casein, 20 nM AlexaFluor488-palloidin labeled actin filaments in M-Buffer, again with 2 mg mL^−1^ casein, M-Buffer alone, and then 100 μL reaction mixtures containing 2 nM Myo19-decorated Qdots, 20 μM CaM, and 0.2 mg mL^−1^ casein in M-Buffer with M-Additives. The effect of calcium was assayed by adding 1.1 mM CaCl_2_ to yield 0.1 mM free Ca^2+^ buffered by 1 mM EGTA in the M-Buffer. After being sealed with silicon grease, chambers were mounted onto the total internal reflection fluorescence microscope where they were imaged immediately at 37 °C. Filament and Qdot velocities were measured using the MTrackJ ImageJ plugin. Qdot run lengths were extracted from the velocity analysis and included even if the Qdots reached the end of an actin filament. Frequency was calculated by the total number of runs divided by the time and the total length of the actin in the field, which was estimated by the average of three measurements (length/sample area ∗ total area).

### Copurification assays

Constructs for expressing individual IQs with N-terminal MBP-TEV and C-terminal polyHis tags were cotransformed with either CaM or RLC12B (untagged) expression plasmids to Rosetta cells, and expression was induced as described previously. The sequences selected for each IQ motif were (IQ1) EQCARCIQGGWRRHRHREQER, (IQ2) WRAVMLIQAAIRSWLTRKHIQ, and (extended IQ3) HAAATVIKRAWQKWRIRMACLAAKELDGVEEKHFSQAPCS. The TEV-site linker and His tag were immediately adjacent to these sequences. For purification, cell pellets were resuspended in Lysis C buffer (25 mM Tris–HCl [pH 8], 300 mM NaCl, 10 mM imidazole [pH 7.5], and 1 mM EGTA) with 0.5 mM β-mercaptoethanol, 1 mM PMSF, 10 μM leupeptin, and 0.4 mg mL^−1^ benzamidine and lysed by microfluidizer. After clarification (aforementioned), lysate supernatants were loaded onto nickel resin, which was then washed with 6 CV Lysis C (wash 1) followed by 2 CV 30 mM imidazole Lysis C (wash 2) and eluted with 250 mM imidazole Lysis C.

### Graphing and statistical analysis

Graphs and statistics were generated using GraphPad Prism 9.4.1 (GraphPad Software, Inc). *t* Tests were performed to compare gliding motility data, with Welsh’s correction applied for unequal variances. Where appropriate, data in Figure 4 were fit to [agonist] *versus* response (rate = minimum + [C] ∗ (plateau − minimum)/(EC_50_ + [C])) or [inhibitor] *versus* response (rate = plateau + (maximum-plateau)/(1 + [C]/IC_50_)) curves using least squares regression. Individual rates are reported in the *Results* section with SD, whereas fitted values are reported with SE of the mean. One-way ANOVA and Mann–Whitney *U* test were respectively employed for multiple comparisons and non-normally distributed data. To calculate run length constants (λ), cumulative frequency plots of the data in Figure 5*D* were fit to single exponentials following the equation: frequency = (frequency_0_ – plateau) ∗ e^−length/λ^ + plateau.

## Data availability

Data to be shared upon request to E. Michael Ostap (ostap@pennmedicine.upenn.edu). Raw MS data are available at ftp://MSV000090375@massive.ucsd.edu (doi:10.25345/C5Z02ZD5B; username: MSV000090375_reviewer, password: Myo19Reviewers).

## Supporting information

This article contains supporting information.

## Conflict of interest

The authors declare that they have no conflicts of interest with the contents of this article.
