# Supplementary material for: Ensembles of human myosin-19 bound to calmodulin and regulatory light chain RLC12B drive multimicron transport
Source: J Biol Chem. 2023 Jan 13;299(2):102906. doi: 10.1016/j.jbc.2023.102906 (PMC9929473; doi:10.1016/j.jbc.2023.102906)
Supplement: Supplemental Figure Legends [file mmc1.docx]

**Supporting Information**

**Figure S1. GFP-RLC12B localized to mitochondria in Myo19-expressing cells.** Image from **Fig. 2D** repeated (GFP-RLC12B) and the corresponding mitochondria channel (MitoTracker), as well as both channels merged: (green) GFP-RLC12B; (magenta) mitochondria. Bar: 2 µm.

**Figure S2. Copurification of MBP-IQ-6xHis constructs with RLC12B and CaM from** ***E. coli***. Coomassie-stained SDS-PAGE gels showing purification steps of MBP-IQs co-expressed and purified with (top) RLC12B and (bottom) CaM. These gels were cropped in **Fig. 3C**. Detail in *Experimental Procedures*.
