# Supplementary figures and images for: Ensembles of human myosin-19 bound to calmodulin and regulatory light chain RLC12B drive multimicron transport
Source: J Biol Chem. 2023 Jan 13;299(2):102906. doi: 10.1016/j.jbc.2023.102906 (PMC9929473; doi:10.1016/j.jbc.2023.102906)

MitoTracker

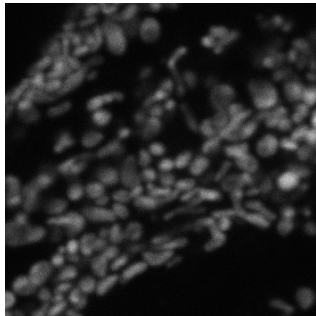

GFP-RLC12B

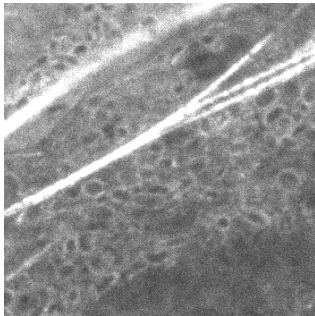

Merge

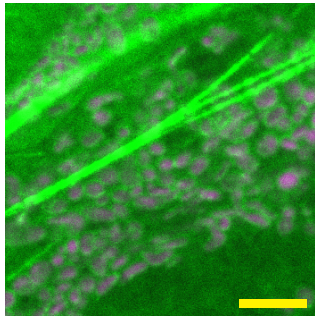

Supplement: Supplemental Figure S1 [file mmc2.pdf]

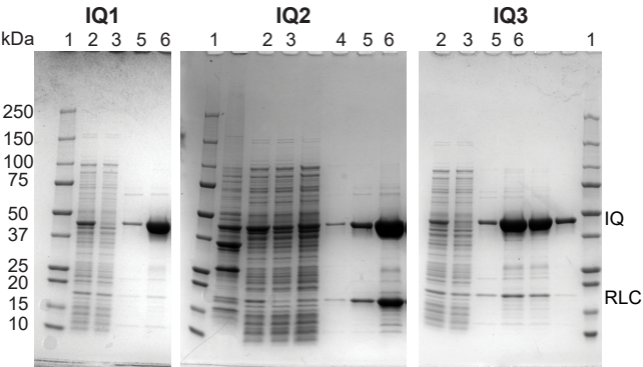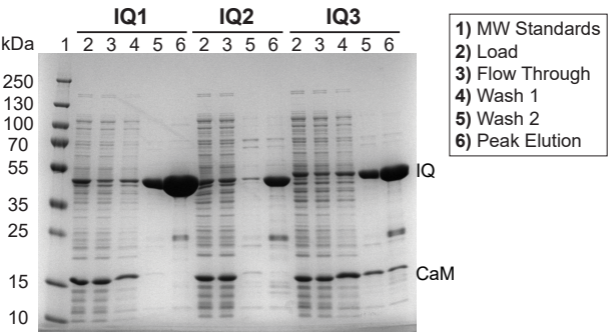

Supplement: Supplemental Figure S2 [file mmc3.pdf]
